# Association of growth rate with hormone levels and myogenic gene expression profile in broilers

**DOI:** 10.1186/s40104-017-0170-8

**Published:** 2017-05-05

**Authors:** Yingping Xiao, Choufei Wu, Kaifeng Li, Guohong Gui, Guolong Zhang, Hua Yang

**Affiliations:** 1 Institute of Quality and Standards for Agro-products, Zhejiang Academy of Agricultural Sciences, Hangzhou, 310021 China; 2 College of Life Sciences, Huzhou University, Huzhou, 313000 China; 3 Department of Animal Science, Oklahoma State University, Stillwater, Oklahoma 74078 USA

**Keywords:** Breast muscle, Broiler, Growth performance, Hormone, Myogenic gene expression

## Abstract

**Background:**

The growth rate often varies among individual broilers of the same breed under a common management condition. To investigate whether a variation in the growth rate is associated with a difference in hormone levels and myogenic gene expression profile in broilers, a feeding trial was conducted with 10,000 newly hatched Ross 308 chicks in a commercial production facility under standard management. At 38 d of age, 30 fast-, 30 medium-, and 30 slow-growing broilers were selected among 600 healthy male individuals. The levels of insulin-like growth factor-1 (IGF-1), triiodothyronine (T3), thyroxine (T4), and growth hormone in the serum or breast muscle were assayed by ELISA or RIA kits, and the expression levels of several representative pro- and anti-myogenic genes in the breast muscle were also measured by real-time PCR.

**Results:**

Results showed that both absolute and relative weights of the breast muscle were in linear positive correlations with the body weight of broilers (*P* < 0.001). Fast-growing broilers had higher concentrations of IGF-1 than slow-growing broilers (*P* < 0.05) in both the serum and breast muscle. The serum concentration of T3 was significantly higher in fast-growing birds than in slow-growing birds (*P* < 0.05). However, no difference was observed in growth hormone or T4 concentration among three groups of birds. Additionally, a decreased expression of an anti-myogenic gene (*myostatin*) and increased expressions of pro-myogenic genes such as *myogenic differentiation factor 1, myogenin, muscle regulatory factor 4, myogenic factor 5, IGF-1*, and *myocyte enhancer factor 2B, C,* and *D* were observed in fast-growing broilers (*P* < 0.05), relative to slow-growing broilers.

**Conclusions:**

Collectively, these findings suggested that the growth rate is linked to the hormone and myogenic gene expression levels in broiler chickens. Some of these parameters such as serum concentrations of IGF-1 and T3 could be employed to breed for enhanced growth.

## Background

Muscle growth, also known as myogenesis, is a complicated but precisely regulated developmental process. Myogenic regulatory factors (MRF) and myocyte enhancer factor 2 (MEF2) proteins are key transcription factors that are positively involved in the regulation of skeletal muscle development [1]. The MRF family of transcription factors is comprised of a group of basic helix-loop-helic proteins such as myogenic differentiation factor 1 (MyoD1), myogenic factor 5 (Myf5), myogenin, and MRF4, whereas the MEF2 family consists of four members denoted as MEF2A, B, C, and D. On the other hand, myostatin (MSTN), a member of the transforming growth factor β (TGF-β) superfamily secreted from skeletal muscle, acts as a potent negative regulator of muscle differentiation and growth [2]. MSTN modifies the muscle fiber-type composition by regulating MyoD and MEF2 expression [3]. Inhibition of MSTN causes myofibre hypertrophy [4], while MSTN over-expression decreases the skeletal muscle mass and fiber size [5].

Additionally, a number of hormones are known to impact on animal growth. The major hormones required to support normal growth in chickens are growth hormone (GH), 3,5,3´-triiodothyronine (T3), thyroxine (T4), and insulin-like growth factor-1 (IGF-1) [6]. IGF-1 have been shown to stimulate the growth of the skeletal muscle by enhancing the rate of protein synthesis and therefore, the concentration of IGF-1 is often positively correlated with the body weight (BW) in broiler chickens [6–8].

It is well known that the growth rate of individual broilers of the same breed exhibits a normal Gaussian distribution under a common management condition. However, it remains elusive about whether there is a difference between fast- and slow-growing broilers in growth-related hormone and myogenic gene expression levels. The objective of the present study was to investigate the association of the growth rate with the concentrations of growth-related hormones in the circulation as well as both pro- and anti-myogenic gene expression levels in the breast muscle of broilers.

## Methods

### Animals

A flock of 10,000 day-of-hatch Ross 308 broiler chicks were raised in a commercial production facility under standard management, with a 24-h photoperiod and 32-34 °C in the first two days, followed by a 16-h photoperiod and a reduction by 2-3 °C per week to a final temperature of 20 °C. The broilers were allowed ad libitum access to water and commercial mash feed. At 38 d of age, 600 healthy male broilers were randomly picked and weighed, from which 30 broilers of the highest, medium, and lowest BW were selected as H, M, and L groups, respectively.

### Sampling

Ten birds were randomly selected from each group. Blood (4 mL each) was collected from the wing vein and centrifuged at 3,000 × *g* and 4 °C for 10 min to obtain serum, which was stored at -80 °C for further analysis. Chickens were then killed by cervical dislocation. The entire breast muscle (including pectoralis major and minor) was weighed after removal. Approximately 3 g of each muscle sample was immediately snap frozen in liquid nitrogen and stored at -80 °C for further analysis as described below.

### Tissue preparation and hormone concentration measurement

The breast muscle samples were homogenized (1:19, w/v) in chilled physiological saline. After centrifuging at 4 °C and 5000 × *g* for 10 min, the supernatants were collected for subsequent assay for various hormones. The IGF-1 concentrations in the serum and muscle were measured with a commercial chicken-specific ELISA kit following the manufacturer’s protocols (Jiancheng Bioengineering, Nanjing, China). Serum concentrations of GH, T3 and T4 were measured with the respective commercial RIA kits (Beijing North Institute of Biological Technology, China).

### Extraction and quantification of total DNA and proteins

The breast muscle was homogenized in chilled physiological saline (1:10, w/v). DNA and proteins were extracted from the muscle homogenates as described [9]. The concentrations of DNA were measured using a NanoDrop1000 Spectrophotometer (NanoDrop Products, Wilmington, DE), and the protein concentrations were measured with a protein assay kit (Jiancheng Bioengineering, Nanjing, China). The percentages of proteins and nucleic acids in each breast muscle sample were calculated.

### RNA extraction and quantitative real-time RT-PCR

Total RNA was extracted from the breast muscle using RNAiso (Takara Bio, Dalian, Liaoning, China). The first-strand cDNA was synthesized using the SuperScript® III Reverse Transcriptase with random primers and an RNase inhibitor (Invitrogen, Carlsbad, CA, USA) as per the manufacturer’s instructions. Gene-specific primers for *IGF-1*, *MSTN*, *MyoD1, myogenin, MRF4, Myf5, MEF2A, MEF2B, MEF2C*, and *MEF2D* were designed using Primer Premier 6.0 (Premier, Ontario, Canada) (Table 1). PCR was performed on the ABI 7500 Real-Time PCR Detection System (Applied Biosystems, Foster City, CA, USA) using SYBR Premix Ex Taq II Kit (Takara Bio) and 40 cycles of 95 °C for 15 s and 60 °C for 30 s. All measurements were performed in triplicate. The fold difference was calculated using the ∆∆Ct method [10, 11] using the geometric means of 18S rRNA and *glyceraldehyde 3-phosphate dehydrogenase* (*GAPDH*) mRNA for data normalization.

**Table 1:** Primers Used for Real-Time PCR

Gene	GenBank Accession Number	Primer Sequence (5' to 3')	Product Size, bp
*IGF-1*	NM_001004384	GAGCTGGTTGATGCTCTTCAGTT	148
		CCAGCCTCCTCAGGTCACAACT	
*MSTN*	NM_001001461	CGCTACCCGCTGACAGTGGAT	132
		CAGGTGAGTGTGCGGGTATTTCT	
*MyoD1*	NM_204214	CCGACGGCATGATGGAGTACA	131
		GTCGAGGCTGGAAACAACAGAA	
*Myogenin*	D90157	GGAGGCTGAAGAAGGTGAACGA	127
		CTCTGCAGGCGCTCGATGTACT	
*MRF4*	D10599	CAGGCTGGATCAGCAGGACAA	106
		GCCGCAGGTGCTCAGGAAGT	
*Myf5*	NM_001030363	CAGAGACTCCCCAAAGTGGAGAT	106
		GTCCCGGCAGGTGATAGTAGTTC	
*MEF2A*	NM_204864	CGGAGGACAGATTCAGCAAACTA	109
		GACACTGGAACCGTAACCGACAT	
*MEF2B*	XM_430389	CACGCCATCAGCATCAAGTCA	156
		GGGGTAGCCCTTGGAGTAGTCAT	
*MEF2C*	XM_001231661	GCCGTCTGCCCTCAGTCAACT	137
		GGGTGGTGGTACGGTCTCTAGGA	
*MEF2D*	NM_001031600	GTGTCTCCCAAGCGACTCACTCT	109
		GTGTTGTATGCGGTCGGCAT	
*GAPDH*	NM_204305	GCCACACAGAAGACGGTGGAT	86
		GTGGACGCTGGGATGATGTTCT	
*18S*	AF173612	CCGGACACGGACAGGATTGACA	94
		CAGACAAATCGCTCCACCAACTAAG	

### Statistical analysis

All data were presented as means ± standard error of the mean (SEM). Statistics were performed using one-way ANOVA using the SPSS software, version 16.0 (Chicago, IL, USA). The differences were considered to be significant, if *P* < 0.05.

## Results

### Body weight and the breast muscle weight

**Fig. 1:**
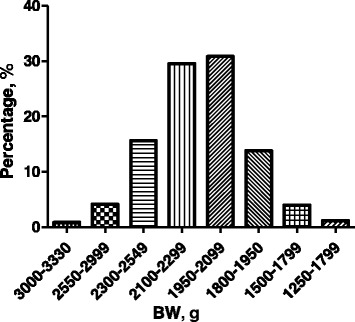
Body weight (BW) distribution of broilers. The data were based on 600, 38-day-old male broilers that were randomly picked and weighed from a group of 10,000 Ross 308 chickens raised under standard management in a single commercial house

**Fig. 2:**
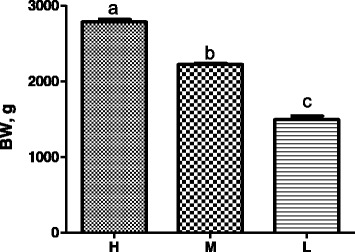
Average body weight (BW) of three groups of broilers. Each bar represents mean ± standard error (SE) of thirty, 38-day-old male broilers per group. Means with no common letter differ significantly (*P* < 0.05). H = fast-growing broilers (heavy BW); M = medium-growing broilers (average BW); L = slow-growing broilers (low BW)

**Fig. 3:**
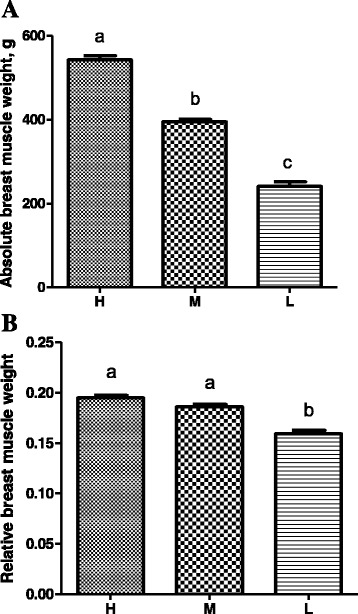
Absolute (**a**) and relative breast muscle weight (**b**) of three groups of broilers. The relative breast muscle weight is the ratio of absolute breast muscle weight to BW. Each bar represents mean ± standard error (SE) of thirty, 38-day-old male broilers per group. Means with no common letter differ significantly (*P* < 0.05). H = fast-growing broilers (heavy BW); M = medium-growing broilers (average BW); L = slow-growing broilers (low BW)

The BW of 600 male Ross 308 broilers that were randomly picked exhibited a normal Gaussian distribution ranging from 1,250 to 3,330 g with an average of 2,213 g (Fig. 1). Thirty heaviest (H), 30 medium (M), and 30 lightest (L) broilers were further selected. The average BW was 2,784, 2,221, and 1,493 g for the H, M, and L groups, respectively, and the H group weighed nearly twice as much as the L group (Fig. 2). As expected, the absolute breast muscle weight was also obviously different (*P* < 0.001) among three groups, with the H group doubling the weight of the L group (Fig. 3a). However, the differences among the relative breast muscle weight (breast muscle weight: BW ratio) in the H and M groups were significantly higher than that in the L group (*P* = 0.026 between H and L; *P* = 0.045 between M and L) (Fig. 3b).

### Concentrations of total DNA, RNA, and proteins in the breast muscle

Total DNA concentration in the breast muscle of the H group was 17% (*P* = 0.042) and 18% (*P* = 0.045) lower than that of the M and L groups, whereas no difference was observed between the M and L groups (Table 2). On the other hand, the total RNA concentration between the H and M groups showed no difference (*P* = 0.884), but was approximately 42% and 39% higher than that in the L group, respectively (Table 2). No difference was seen with the total protein concentration in the breast muscle among three groups of broilers (*P* = 0.661) (Table 2). The protein:DNA ratio was positively correlated with the BW (*P* = 0.049), and the RNA:protein ratio showed the same trend (*P* = 0.086). The RNA:DNA ratio also exhibited an obvious correlation with the BW among the H, M, and L groups (*P* = 0.006), with the H group being significantly higher than the M group and the M group significantly higher than the L group (Table 2).

**Table 2:** The concentrations of DNA, RNA, and proteins in the breast muscle of broilers (*n* = 10)

Item	H	M	L	SEM	*P*-value
DNA, mg/g	1.68^b^	2.02^a^	2.06^a^	0.07	0.044
RNA, mg/g	3.04^a^	2.97^a^	2.14^b^	0.11	0.036
Protein, mg/g	86.32	88.65	84.97	2.54	0.661
Protein:DNA	49.52^a^	43.87^ab^	39.71^b^	1.61	0.049
RNA:Protein	0.035	0.033	0.025	0.003	0.086
RNA:DNA	1.81^a^	1.47^b^	1.06^c^	0.08	0.006

^a-c^Means in the same row with different superscript letters differ significantly (*P* < 0.05)

H = fast-growing broilers (heavy BW group); M = medium-growing broilers (average BW group); L = slow-growing broilers (low BW group)

### Concentrations of hormones in the serum and breast muscle

**Fig. 4:**
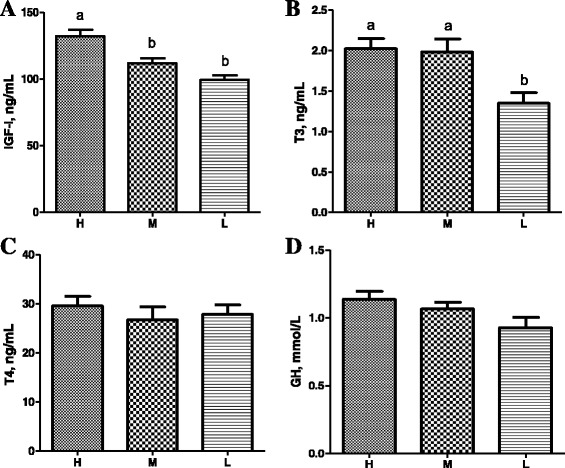
The serum concentrations of fours hormones including IGF-1 (**a**), T3 (**b**), T4 (**c**), and GH (**d**) in broilers. All hormones were measured by chicken-specific ELISA on 38-day-old male broilers. Each bar represents mean ± standard error (SE) of ten replicates. Means with no common letter differ significantly (*P* < 0.05). H = fast-growing broilers (heavy BW); M = medium-growing broilers (average BW); L = slow-growing broilers (low BW). IGF-1, insulin-like growth factor-1; T3, triiodothyronine; T4, thyroxine; GH, growth hormone

**Fig. 5:**
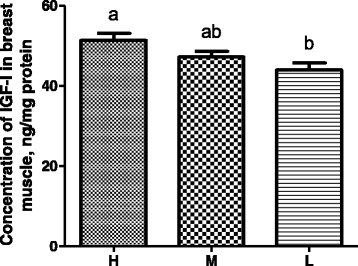
The concentrations of IGF-1 in the breast muscle of broilers. IGF-1 (insulin-like growth factor-1) was measured by chicken-specific ELISA on 38-day-old male broilers. Each bar represents mean ± standard error (SE) of ten replicates. Means with no common letter differ significantly (*P* < 0.05). H = fast-growing broilers (heavy BW); M = medium-growing broilers (average BW); L = slow-growing broilers (low BW)

The serum concentration of IGF-1 showed an obvious linear relationship with the BW (Fig. 4a). The H group had approximately 18% (*P* = 0.044) and 33% (*P* = 0.027) higher IGF-1 concentrations than the M and L groups, respectively. The serum concentration of T3 in the L group was approximately 50% (*P* = 0.017) and 47% (*P* = 0.029) lower than those in the H and M groups, respectively (Fig. 4b). However, no significant difference was observed in the serum concentrations of GH and T4 among the H, M, and L groups (Fig. 4c and d). Similar to the serum concentration, the IGF-1 concentration in the breast muscle was also linearly correlated with the BW (Fig. 5). Comparing with the L group broilers, the IGF-1 concentration in the breast muscle showed a 18% increase (*P* = 0.013) in the H group.

### Expression levels of myogenic genes in the breast muscle

To study the correlation between the expression levels of major myogenic genes and the BW, total RNA was isolated from the breast muscle and subjected to reverse transcription and real-time PCR analysis of the genes for *MyoD1, Myogenin, MRF4, Myf5, MEF2A, MEF2B, MEF2C, MEF2D, IGF-1*, and *MSTN*. As shown in Table 3, the expression levels of all pro-myogenic genes but *MEF2A* showed a significant decrease (*P* < 0.05) in the L group relative to the H group. The pro-myogenic genes, namely *MEF5C*, showed a strong linear correlation (*P* < 0.05) with the BW, with the H group giving the highest expression and the L group the lowest expression. No difference (*P* > 0.05) was observed with *MyoD1, MRF4* or *MEF2B* between the H and M group, whereas *Myogenin* and *MEF2D* showed no difference between the M and L group. As for the expression of *MSTN*, an anti-myogenic gene, the H and M group showed no statistical difference, but both groups were significantly lower than the L group (Table 3). Interestingly, the *IGF-1* mRNA expression level was also significantly reduced (*P* = 0.026), relative to the H or M group (Table 3).

**Table 3:** The relative mRNA levels in the breast muscle of broilers (*n* = 10)

Item	H	M	L	SEM	*P*-value
*MyoD1*	0.84^ab^	1.00^a^	0.66^b^	0.065	0.017
*Myogenin*	2.94^a^	1.00^b^	1.29^b^	0.111	<0.001
*MRF4*	1.22^a^	1.00^a^	0.78^b^	0.078	0.047
*Myf5*	0.85^a^	1.00^a^	0.61^b^	0.076	0.044
*MEF2A*	1.23	1.00	1.14	0.080	0.110
*MEF2B*	1.06^a^	1.00^a^	0.60^b^	0.042	<0.001
*MEF2C*	1.22^a^	1.00^b^	0.74^c^	0.062	0.024
*MEF2D*	1.32^a^	1.00^b^	0.89^b^	0.052	0.015
*MSTN*	1.15^b^	1.00^b^	1.82^a^	0.101	0.041
*IGF-1*	0.94^a^	1.00^a^	0.51^b^	0.060	0.031

^a- c^Means in the same row with different superscript letters differ significantly (*P* < 0.05)

H = fast-growing broilers (heavy BW group); M = medium-growing broilers (average BW group); L = slow-growing broilers (low BW group)

## Discussion

Genetic differences among the chicken breeds of different growth rates have been extensively studied and a number of genes and quantitative trait loci have been reported in controlling the growth rate of chickens [12–15]. However, little is known regarding genetic and hormonal variations among individual broilers of the same breed under a common management. This study demonstrated that there is a large variation in the growth rate among individual birds raised under industrial standard management practices, with BW ranging from 1,250 g to 3,330 g. To elucidate the mechanism of the BW variations in a cohort of broiler chickens, the serum concentrations of GH, T3, T4, and IGF-1 were measured in the present study. The result showed that the T3 serum concentration was higher in heavier broilers than lighter ones, with no difference seen with the T4 concentration. This is consistent with an earlier report that the plasma concentration of T3, but not T4, was reduced in the sex-linked dwarf chickens relative to the normal control breed [16]. Broilers with heavier BW had a higher serum concentration of IGF-1 in our study, which is in agreement with an earlier observation that a reduction in the growth rate was associated with a decrease in circulating concentrations of IGF-1 in chickens [6]. Thus, higher levels of T3 and IGF-1 in the circulation accelerate growth in broilers and can be good predictors of faster growth.

Muscle growth is known to be accelerated by activation of the IGF-1 signaling pathway [8, 17], which is consistent with our observation that IGF-1 in the breast muscle were higher at both protein and mRNA levels in fast-growing than slow-growing broilers, which is probably due to increased methylation in the promoter region of the *IGF-1 receptor* gene in slow-growing broilers [18].

Besides *IGF-1*, altered expressions of multiple myogenic genes was also observed between heavy and light broilers in this study. Pro-myogenic genes such as *MyoD1* and *Myf5* are important in the formation of skeletal muscle [19, 20]. Other pro-myogenic genes such as *myogenin* and *MRF4* are directly involved in the differentiation of myotubes [21]. Consistently, we observed a significant increase in the expression of *MyoD1, Myf5, myogenin*, and *MRF4* in fast-growing birds relative to slow-growing ones. Similarly, three members of the pro-myogenic MEF2 family, namely *MEF2B, MEF2C* and *MEF2D*, were also significantly up-regulated in fast-growing broilers. On the other hand, the anti-myogenic *MSTN* mRNA levels are significantly lower in heavy broilers than light ones.

The skeletal muscle is a heterogeneous tissue composed of individual muscle fibers, diversified in size, shape and contractile protein content with different morphology, metabolism, and physiology [22]. Muscle mass can be affected by alterations in satellite cell activities, which dictate mature muscle size [22]. In the present study, fast-growing broilers had a greater breast muscle yield than slow-growing ones, reminiscent of previous studies [8, 23]. It is well known that DNA contents, protein:DNA and RNA:DNA ratios reflect cell population, cell size, and cellular metabolic activities, respectively [24]. An increase in the amount of total cellular RNA is likely due to enhanced synthetic activities [9]. In this study, increased protein:DNA and RNA:DNA ratios and total RNA content, accompanied with a decrease in the DNA content, in the breast muscle of fast-growing broilers suggested that heavier birds has less muscle cells but higher cellular activities. The results coincide with previous studies showing that heavier chickens had greater muscle mass with more satellite cells with enhanced proliferative activities [23, 25].

Hatching weight was shown to be a primary predictor of the market weight in chicks. The average BW of broilers at 42 d of age with heavy (48.3 g) and light hatching weight (41.7 g) became 2,368 g and 2,116 g, respectively [8]. Similarly, broilers on d 41 hatching at 53.1 g was about 1.1-fold heavier that those hatching at 43.5 g [23]. It is important to note that, although the breast muscle weight differs significantly between heavy and light broilers, the abdominal fat content shows no difference at marketing [23], suggesting that the genes involved in adipogenesis may not play a major role in the big variation in BW seen in a commercial broiler production facility.

In this study, we observed a significant difference in both mRNA and protein levels of IGF-1 in the breast muscle and circulation between fast- and slow-growing chickens on d 38. In fact, such a difference in the IGF-1 protein concentration is also evident on day of hatch between the broilers with heavy hatching weight and the ones with light hatching weight [23]. Consistently, IGF-1 levels are positively associated with growth rate in broiler strains divergently selected for high or low growth potential [26, 27], suggesting that IGF-1 can be a good predictor for growth [28]. However, the heritability of IGF-1 is only 0.1 [29] and therefore, its potential might still be limited as a useful parameter for future broiler breeding for enhanced performance. Nevertheless, to our knowledge this is the first study to reveal a positive correlation between IGF-1 and growth rate in a large population of broilers growing under standard commercial management.

It is noted that the serum concentration of T3 was also observed to be significantly different between fast- and slow-growing broilers on d 38 in our study. It will be important to examine a possible difference in its concentration on d 0 in order to explore the growth predictive value of T3.

## Conclusions

A variation in the growth rate among individual broilers of the same breed is caused by an alteration in the growth-related hormone and expression of the genes involved in myogenesis. Serum concentration of IGF-1 and T3 is positively correlated with the growth rate of broilers. Most pro-myogenic genes such as *MyoD1, Myogenin, MRF4, Myf5, MEF2B, MEF2C*, and *MEF2D* are also associated positively with the growth, while *MSTN*, an anti-myogenic gene, shows a negative association. Some of these parameters may have potential to be further explored for the breeding purpose.

## Abbreviations

BW: body weight
GH: growth hormone
IGF-1: insulin-like growth factor-1
MEF2: myocyte enhancer factor 2
MRF: myogenic regulatory factors
MSTN: myostatin
Myf5: myogenic factor 5
MyoD1: myogenic differentiation factor 1
T3: triiodothyronine
T4: thyroxine
